# An easy method for developing fusion enabled SARS-CoV2 virus fusion mimic (SCFM), bypassing the need of Bio Safety Level (BSL) facility

**DOI:** 10.1080/21655979.2021.1955509

**Published:** 2021-08-26

**Authors:** Abhishek Das, Satarupa Dutta, Dewanshu Sharma, Amit Pal, Nirmalya Ganguli, Subeer S. Majumdar

**Affiliations:** Gene and Protein Engineering Laboratory, National Institute of Animal Biotechnology, Hyderabad, India

**Keywords:** COVID19, SARS-CoV2, S protein, syncytia, fusion, fusion mimic

## Abstract

Widespread infection due to severe acute respiratory syndrome coronavirus 2 (SARS-CoV2) has led to a global pandemic. Currently, various approaches are being taken up to develop vaccines and therapeutics to treat SARS-CoV2 infection. Consequently, the S protein has become an important target protein for developing vaccines and therapeutics against SARS-CoV2. However, the highly infective nature of SARS-CoV2 restricts experimentation with the virus to highly secure BSL3 facilities. The availability of fusion-enabled, nonreplicating, and nonbiohazardous mimics of SARS-CoV2 virus fusion, containing the viral S or S and M protein in their native conformation on mammalian cells, can serve as a useful substitute for studying viral fusion for testing various inhibitors of viral fusion. This would avoid the use of the BSL3 facility for fusion studies required to develop therapeutics. In the present study, we have developed SARS-CoV2 virus fusion mimics (SCFMs) using mammalian cells transfected with constructs coding for S or S and M protein. The fusogenic property of the mimic(s) and their interaction with the functional human ACE2 receptors was confirmed experimentally. We have also shown that such mimics can easily be used in an inhibition assay. These mimic(s) can be easily prepared on a large scale, and such SCFMs can serve as an invaluable resource for viral fusion inhibition assays and in vitro screening of antiviral agents, which can be shared/handled between labs/facilities without worrying about any biohazard while working under routine laboratory conditions, avoiding the use of BSL3 laboratory.

**Abbreviations :**SCFM: SARS-CoV2 Virus Fusion Mimic; ACE2: Angiotensin-Converting Enzyme 2; hACE2: Human Angiotensin-Converting enzyme 2; MEF: Mouse Embryonic Fibroblasts; HBSS: Hanks Balanced Salt Solution; FBS: Fetal Bovine Serum

## Graphical abstract

**Figure:**
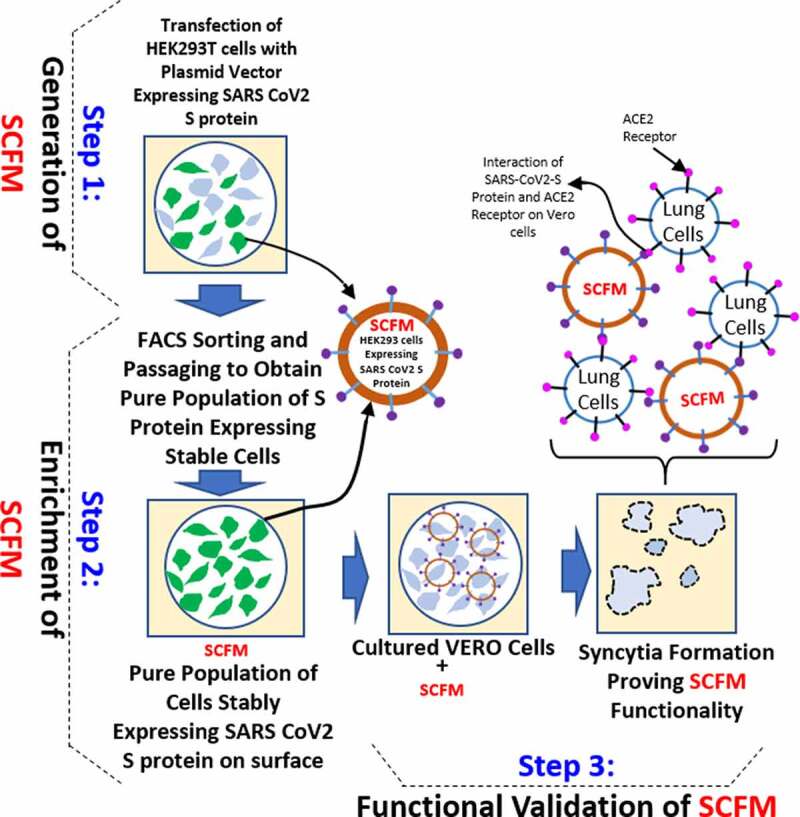

## 1. Introduction

The disease caused by the infection of severe acute respiratory syndrome coronavirus 2 (SARS-CoV2) has been termed as Coronavirus disease 2019 (COVID-19) [1]. Among all the structural proteins of the SARS-CoV2, the S protein is the major one and plays a crucial role in the viral attachment with the target cells through a specific interaction of its ligand. Such an interaction initiates the process of viral fusion/infection as well as immune responses [2–4]. However, due to the highly infective nature of SARS-CoV2, the handling and experimentation using this virus must be carried out in the biosafety level 3 (BSL3) facility [5]. The establishment of such a BSL3 facility is time consuming and involves huge costs. In most laboratories, people are forced to work with pseudoviruses [6] or heat-inactivated/dead viruses worldwide. These constraints have restricted the development of effective therapeutics against the COVID-19 at a fast pace [7,8].

In this scenario, it would be better if a noninfectious cell line can be generated expressing SARS-CoV2 virus S or S and M protein, both in functional conformation on their membrane, which would be a fusion-enabled, nonreplicating, and nonbiohazardous substitute.

The lipid bilayer structured mammalian cell membrane [9] can be used for mimicking the viral fusogenic property if studded with additional viral proteins that help in interaction followed by fusion with host cells. Previously, we developed a nonreplicating virosome consisting of Sendai virus membrane that can fuse with mammary epithelial cells based on the specific interaction of receptor present on the viral membrane with its ligand on mammary epithelial cells for the delivery of transgene [10]. Here, we report the generation of fusion mimics of SARS-CoV2 virus based on cultured mammalian cells, which is fusion enabled with its specific target cells like SARS-CoV2 virus, through specific receptor–ligand interaction. This method will be useful in generating such mimics for other types of animal viruses that also requires a BSL3 facility for its handling and usage.

In this study, we hypothesized that mammalian cells stably expressing the SARS-CoV2 S protein or S and M protein together can mimic the fusogenic membrane property of the SARS-CoV2 virus. We aimed to generate plasmid vectors expressing S or M protein in mammalian cells, which can fuse with its target cells through the interaction of such membrane-localized S protein with its receptor ACE2. If successful, such a stable cell line, SARS-CoV2 virus fusion mimics (SCFMs), can be used as a platform for testing various antiviral agents, which can be screened on a large scale at a very fast pace without the need of a BSL3 facility.

## 2. Materials and methods

### 2.1. Cell lines

HEK293T (ATCC® CRL-3216™), MCF7 (ATCC® HTB-22™), and VERO (ATCC® CCL-81™). HEK293T, MCF7, VERO cells were obtained from the ATCC and cultured in Dulbecco’s Modified Eagle’s Medium (DMEM) High Glucose supplemented with 10% Fetal Bovine Serum (FBS) and kept in an incubator at 37 °C in 5% CO_2_ in humidified condition.

### 2.2. Isolation and culture of mice embryonic fibroblast cells

Primary mouse embryonic fibroblasts (MEFs) were isolated from 13.5-day embryos. Embryos were harvested, followed by the removal of head and liver from the embryos. The remaining embryonic tissue was minced using a scalpel blade and digested using 0.25% trypsin for 10 min in a shaking water bath. The cells were washed two to three times using Hank’s Balanced Salt Solution (HBSS) and seeded in culture dishes using DMEM high glucose supplemented with 10% FBS.

### 2.3. Plasmid constructs

pIRES2-EGFP mammalian expression plasmids were procured from Clontech. pCAG-DsRed2 (Addgene plasmid no. – 11,151) and mTagRFP-Membrane-1 (Addgene plasmid no. – 57,992) mammalian expression plasmids were procured from Addgene. Human codon-optimized cDNA of S protein gene (pUC57-2019-nCoV-S, Human; MC_0101081) and native M protein gene (pET-28a (+)-M Protein; MC_0101136) of SARS-CoV2 were procured from GenScript and further used for molecular cloning experiments. pK18-hACE2 plasmid construct was shared by Dr. Sudhanshu Vrati, Regional Centre for Biotechnology, New Delhi. **Generation of pIRES2-DsRed2 mammalian expression plasmid**: PCR amplification of *DsRed2* gene from pCAG-DsRed2 plasmid using specific primers (Table 1) was performed, followed by the removal of EGFP gene from pIRES2-EGFP plasmid by another PCR amplification using vector backbone-specific primers (Table 1). Ligation of both the fragments was performed by blunt-end cloning method as mentioned in Sambrook et. al. [11] to clone *DsRed2* gene to pIRES2 vector backbone to generate pIRES2-DsRed2 mammalian expression plasmid. **pCMV-SARSCoV2S-IRES2-EGFP mammalian expression construct preparation**: Sub-cloning of S protein cDNA from pUC57-2019-nCoV-S GenScript plasmid to pIRES2-EGFP mammalian expression vector under ubiquitous CMV promoter was performed by digestion using SacI and XmaI restriction enzymes followed by staggered end ligation method as mentioned in Sambrook et. al [11]. This was further confirmed by sanger sequencing. **pCMV-SARSCoV2M-IRES2-DsRed2 mammalian expression construct preparation**: PCR amplification of native M protein cDNA from pET-28a (+)-M protein plasmid using specific primers (Table 1) was performed to incorporate SacI restriction enzyme site at 5ʹ-end and XmaI restriction enzyme site at 3ʹ-end of native M protein cDNA followed by sticky end cloning method as mentioned in Sambrook et. al. [11] to clone native M protein cDNA into newly generated pIRES2-DsRed2 mammalian expression vector. This was further confirmed by sanger sequencing.

**Table 1.** List of Primers used in this study

Sr. No.	Primer ID	Primer Sequence (5ʹ – – -3ʹ)	Annealing Temperature (°C)	Description
1	DsRed2_FP	GCCTCCTCCGAGAACGTCAT	59	for generation of pIRES2-DsRed2 mammalian expression plasmid
2	DsRed2_RP	CTACAGGAACAGGTGGTGGC	59	
3	pIRES2-EGFP_FP	TAAAGCGGCCGCGACTCTAG	56	
4	pIRES2-EGFP_RP	CATGGTTGTGGCCATATTATC	56	
5	2019-nCoV_M_FP	ATAGAGCTCGCCACCATGGCA GATTCCAACGGTAC	65	for pCMV-SARSCoV2M-IRES2-DsRed2 mammalian expression construct preparation
6	2019-nCoV_M_RP	CCGCCCGGGTTACTGTACAAG CAAAGCAATATTGT	65	
7	2019-nCoV_S_qRT_FP	ATTGTACCGAGGTGCCCGTG	60	for detection of S-protein at genomic as well as transcript level
8	2019-nCoV_S_qRT_RP	AGGCATCCGGCTCTTGTCTG	60	
9	2019-nCoV_M_qRT_FP	TGTGACATCAAGGACCTGCC	60	for detection of M-protein transcripts
10	2019-nCoV_M_qRT_RP	CTGAGTCACCTGCTACACGC	60	
i	ACE2_qRT_FP	AAAGTGGTGGGAGATGAAGC	60	for detection of ACE2 transcripts
12	ACE2_qRT_RP	GTTTCATCATGGGGCACA	60	
13	ACTB_qRT_FP	GCAAAGACCTGTACGCCAAC	60	for housekeeping genes transcripts
14	ACTB_qRT_RP	GATCCACACGGAGTACTTGC	60	
15	GAPDH_qRT_FP	TGCCCTCAACGACCACTTTG	60	
16	GAPDH_qRT_FP	CTGGTGGTCCAGGGGTCTTA	60	

### 2.4. Transfection

HEK293T cells were cultured in DMEM high glucose containing 10% FBS at 37°C in a humidified atmosphere of 5% CO_2_. Cells were passaged and plated in 12-well plates. When the cells reach 60% confluency, the cells were transfected with pCMV-SARSCoV2S-IRES2-EGFP and pCMV-SARSCoV2M-IRES2-EGFP individually or together (Co-Transfection) using Lipofectamine™ 2000 Transfection Reagent (Invitrogen) in the ratio of 1:2 (DNA: Lipofectamine). The cells transfected with pCMV-SARSCoV2S-IRES2-EGFP alone were named SCFM1. Co-transfected cells were named SCFM2. 24 h post transfection, the cells were visualized under UV with different filters in ZOE™ Fluorescent Cell Imager (Bio-Rad, USA) for assessing the transfection efficiency through marker protein expression. Similarly, VERO, MCF7, and MEF cells were transfected with different constructs following the same protocol.

### 2.5. FACS sorting of transfected cells

HEK293T cells transfected with pCMV-SARSCoV2S-IRES2-EGFP alone (SCFM1) or co-transfected with pCMV-SARSCoV2M-IRES2-EGFP (SCFM2) were harvested and passed through 40 µm cell strainer to get single cell suspension. All the processing steps were performed on ice aseptically. The neutral pH of every solution was maintained to prevent quenching of EGFP fluorescence. The cell suspension was collected in a closed cap FACS tube and was analyzed in BD-FACS Aria III. Initially, the live cell population was gated out from the dead cells, followed by gating of EGFP positive cells and EGFP – DsRed2, dual positive cells out of the total live cell population. These sorted cells were further cultured as described in the previous sections.

### 2.6. Immuno cytochemistry

Transfected HEK293T cells were grown on coverslips. Cells were fixed with 2% paraformaldehyde for 30 min. For staining in permeabilized condition, cells were permeabilized with 0.1% TRITON X-100 for 1 min, whereas, for staining in nonpermeabilized condition, the cells were not treated with any detergent. Followed by this, the cells were washed with 1 X PBS and were treated with blocking buffer (3% w/v BSA in 1 X PBS) for 30 min at room temperature. The cells were washed 3 times with 1 X PBS and incubated with primary antibody Rabbit polyclonal anti spike glycoprotein (ProSci, catalog No. 3223) in 1:500 dilution prepared in 1% BSA overnight at 4^°^C. The cells were washed thrice with 1 X PBS followed by incubation with secondary antibody (Donkey Anti mouse 546, Invitrogen USA) in a moist chamber at 37^°^C. The coverslips containing cells were washed thrice and counterstained with nuclear stain Hoechst (Sigma Aldrich, catalog No. 14,530). The coverslips were mounted withProLong^TM^ Gold Antifade Mountant (Invitrogen, USA) and imaged under fluorescence microscope Axio Observer (Zeiss, Germany) with different UV filters.

### 2.7. Generation of HEK293T cell stably expressingSARS-CoV2 S protein (SCFM1)

pCMV-SARSCoV2S-IRES-EGFP construct was first linearized by using SfiI restriction enzyme. The column purified linearized pCMV-SARSCoV2S-IRES-EGFP construct was used for stable transfection of HEK293T cells at passage# 3 (P#3) using lipofectamine 3000 transfection reagent. For selecting cells that will stably express SARSCoV2S and EGFP reporter genes, 48 h post transfection, the EGFP expressing cells were FACS sorted and maintained further by passaging till passage #7, upto 16^th^ day post sorting. After that, the cells were processed for a second round of sorting using the same FACS parameters, and the EGFP positive cells were further maintained till passage#10. Yet another round of FACS sorting was performed to select cells stably expressing EGFP and maintained till passage #17. Finally, master and working cell banks were made from these HEK293T cells stably expressing EGFP and SARS-CoV2 S protein.

### 2.8. gDNA isolation and PCR analysis

Genomic DNA was extracted from HEK293T cells stably expressing EGFP and SARS-CoV2 S protein using Hi-Yield Plus Genomic DNA Mini-kit (RBC), following the manufacturer’s guidelines. To confirm the stable integration of the construct, PCR was carried out from gDNA sample using Taq DNA Polymerase (Genei, India) as per manufacturer instruction using forward and reverse primers specific for S-protein (Table 1).

### 2.9. RNA isolation and RT-PCR analysis

RNA was extracted from cells using TRIzol (TRI) reagent (Sigma Aldrich, USA) following the manufacturer’s guidelines. Reverse transcription (RT) reaction was carried out using a two-step PrimeScript cDNA synthesis kit (TaKaRa, USA) using guidelines provided by the manufacturer. PCR was carried out from cDNA using TaqDNA Polymerase (Genei, India) using forward and reverse primers specific for S-protein, M-protein, ACE2, and human Beta-actin and GAPDH, as housekeeping control (Table 1).

### 2.10. Fusion assay

The fusion experiments were performed as per the protocol described by Steinberg et. al [12] with modification. Briefly, the transfected or un-transfected VERO, MCF7, MEF cells, and newly generated SCFM1, were cultured. SCFM1 along with VERO/MCF7/MEF cells were harvested and seeded together in a 12-well plate on top of coverslips in high density. The cells were then left to fuse for 30 hours in an incubator at 37°C with 5% CO_2_. The plates were taken out and the cells were fixed using paraformaldehyde. The coverslips containing cells were washed thrice and counterstained with nuclear stain Hoechst (Sigma Aldrich, catalog No. 14530). The coverslips were mounted withProLong^TM^ Gold Antifade Mountant (Invitrogen, USA). The slides were then visualized under UV with different filters in Zeiss Axio observer fluorescent microscope.

### 2.11. Fusion inhibition assay

The newly generated stable SCFM1 were cultured and maintained for 36 h followed by FACS- based sorting of EGFP positive cells. Upon sorting of the EGFP expressing cells, the cells were divided into two fractions. One fraction of sorted cells were incubated at room temperature for 1 h with SARS-CoV2 Anti spike antibody in the working dilution of 20ngml^−1^, whereas the other fraction was kept as control. The VERO cells were harvested using trypsinization and plated together with the sorted SCFM1. In set 1, both SCFM1 and VERO cells were seeded on the top of coverslips with a cell density of 4 × 10^5^ cells each, in set 2, 4 × 10^5^ SCFM1 cells were plated together with2 × 10^5^ VERO cells whereas, in set 3, 2 × 10^5^ SCFM1 cells were plated together with 2 × 10^5^ Vero cells. The co-cultured cells were maintained for 18h–24h in humidified CO_2_ incubator. After 24 h of co-culture, the cells were fixed with 2% paraformaldehyde, stained with Hoechst dye, and were visualized in Zeiss Axio observer fluorescence microscope to check syncytia formation. We captured multiple images from all 4 quadrants of the well. All the images were analyzed and syncytia were counted using Zen 3.1 lite software. Graph was plotted with the mean of syncytia formed with and without inhibition using statistical analysis.

### 2.12. Statistical analysis

The statistical analysis was performed using Graph Pad Prism Software. Graph of inhibition assay was generated using unpaired nonparametric Student's t-test analysis of the total syncytia counted from with and without inhibitor treated experiment set.

## 3. Results and discussion

SARS-CoV2 has led to a global crisis. The highly infective nature of SARS-CoV2 demands the use of the BSL3 facility for experimentation with this virus for the development of therapeutics or testing of vaccine efficacy. The availability of fusion-enabled, nonreplicating and nonbiohazardous SCFMs containing the viral S or S and M protein in their native conformation can serve as a useful substitute for studying viral fusion and testing various inhibitors of viral fusion to use them for therapeutic purposes under regular laboratory conditions without the risk of infection. In this study, we hypothesized that mammalian cells stably expressing the SARS-CoV2 S protein or S and M protein together can mimic the fusogenic property of the SARS-CoV2 virus. We aimed to generate plasmid vectors expressing S or M protein in mammalian cells, which can fuse with its target cells through the interaction of membrane localized S protein with its receptor ACE2. We have developed SCFMs using cultured mammalian cells. The fusogenic property of the mimic(s) and its interaction with the functional human ACE2 receptor were confirmed experimentally. We have also shown that such mimics can easily be used in an inhibition assay. Such stable cell lines,i.e., SCFMs, can be used as a platform for testing various antiviral agents, which can be screened on a large scale at a very fast pace without the need for a BSL3 facility.

**Figure 1.**
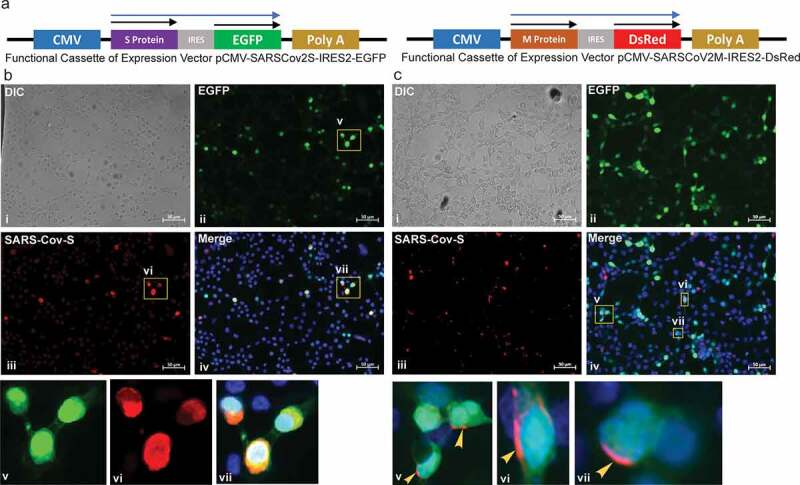
Image showing construct map of the expression cassette and expression of SARS-CoV2 S protein on the surface of SCFM1, detected by Immunocytochemistry analysis

We designed two different mammalian expression vectors that expressed the S and M proteins of SARS-CoV2 virus independently. Human codon-optimized cDNA of S protein and cDNA of M protein in native form were procured and subcloned into pIRES2-EGFP and pIRES2-DsRed2 expression vectors, respectively. Both the cDNAs were placed individually in a bicistronic expression cassette under the control of Cyto-megalo virus immediate early promoter (CMV) followed by IRES sequence and cDNA of either *egfp* or *DsRed2*, to generate plasmids pCMV-SARSCoV2S-IRES2-EGFP and pCMV-SARSCoV2M-IRES2-DsRed2 respectively (Figure 1(a) and S1a). The presence of IRES sequence in these constructs ensures coding of viral S or M protein separately from marker protein at the translational level [13].

(a) Diagram showing the functional expression cassette of the plasmid construct bearing cDNA of SARS-CoV2 S and M protein. The **Blue arrow** indicates the extent and direction of mRNA expression. **The black arrow** indicates the extent and direction of individual Open Reading Frame (ORF) to be coded separately.(b) HEK293T cells showing expression of SARS-CoV2 S protein in the cytoplasm. The expression of EGFP was detected throughout the cytoplasm along with the expression of SARS-CoV2 S protein. The colocalization of both the protein was prominent. **i**. Represent the phase-contrast images. **ii**. Shows the image captured under UV illumination with FITC filter (for EGFP). **iii**. Shows the image captured under UV illumination with TRITC filter (for AF546). **iv**. Shows the merged images. **v., vi.**, and **vii**. show the magnified view of the area marked in the image **iv**. The nucleus was stained with Hoechst, captured under UV illumination with Blue filter. Scale bar 50 µm.(c) HEK293T cells showing expression of SARS-CoV2 S protein on the surface. The expression of EGFP was detected throughout the cytoplasm. The expression of SARS-CoV2 S protein was detected specifically on the surface of the plasma membrane only, counterstained with AF546, and stood distinctly separate from cytoplasmic EGFP expression, marked by a **yellow arrowhead. v., vi**. and **vii**. shows the magnified view of the area marked in image **iv**. The nucleus was stained with Hoechst. Scale bar 50 µm.

We selected HEK293T cells for the generation of such SCFMs by studding the functional S and M protein on its membrane through transfecting it with mammalian expression vectors carrying cDNA of S and M protein. Expression of EGFP and DsRed2 was detected in the cells transfected with S and M protein-expressing construct, respectively (Figure S1b) as compared to un-transfected cells (Figure S1c). The expression of mRNA of S and M was confirmed by RT-PCR analysis in the transfected HEK293T cells as compared to un-transfected cells (Figure S1d). These experiments together confirmed the functionality of the newly generated plasmid vectors.

We detected the expression of S protein by immunocytochemical (ICC) analysis of the cells using an antibody specific to SARS-CoV2 S protein after permeabilization of the cell membrane by detergent treatment. We detected the expression of S protein in the transfected cells, specifically as compared to un-transfected cells (Figure 1(b) and S2a). For effectively mimicking the arrangements of the structural protein of the SARS-CoV2 virus on mammalian cells, the presence of Receptor Binding Domain (RBD) of the major structural protein S on the extracellular surface of the membrane is necessary. It has been shown that the extracellular domain of S glycoprotein interacts with the ACE2 receptor, and its interaction is known to induce conformational changes in the host cell required for membrane fusion [14,15]. To confirm that the expressed S protein is localized on the extracellular side membrane and the RBD is exposed on the extracellular surface, we performed ICC analysis of the pCMV-SARSCoV2S-IRES2-EGFP transfected cells in a nonpermeabilized condition. In a nonpermeabilized state, the antibody could not access the cytoplasmic fraction of the expressed S protein and could only bind and detect the membrane-bound fraction. The expression of S protein was seen on the surface of the membrane (Figure 1(c) and S2b) generating a hope that the RBD will be available for subsequent interaction with its ligand, the ACE2 receptor, followed by effective membrane fusion of this newly developed SCFM [15].

Upon confirmation of the expression of S protein on the membrane, we proceeded further for the enrichment of such cells that can work as a mimic. We transfected HEK293T cells either by construct expressing only S protein (SCFM1) or together with a construct expressing M protein (SCFM2). We sorted both the cell types using Fluorescence Activated Cell Sorter (FACS), based on the expression of marker protein EGFP and DsRed2. SCFM1 showed EGFP fluorescence, while SCFM2 showed EGFP as well as DsRed2 fluorescence. Both the population of cells (EGFP positive and EGFP + DsRed2 positive) were re-cultured and detected retained expression of the marker protein in both the SCFM (Figure S3a). All further studies were continued with SCFM1 as M protein is known to have no role in the fusogenic property of the SARS-CoV2 virus [16]. We proceeded for the generation of SCFM1 that retained the expression SARS-CoV2 S protein stably on their surface. To obtain this, we performed transfection followed by selecting the EGFP positive HEK293T cell population in continuous passaging. At passage 14^th,^ we obtained ~17% EGFP positive cells (Figure S3b) which were further enriched and maintained until passage #17 followed by cryopreservation for further use. These cells at passage #17 showed enhanced EGFP expression (Figure S3c). To confirm the stable integration of the construct, we performed PCR from the gDNA isolated from these cells. In PCR analysis, we detected SARS-CoV2 S expressing plasmid construct in this newly developed stable SCFM1 compared to wild type HEK293T cells (Figure S3d). This SCFM1 also showed strong EGFP expression and expression of the transcript of SARS-CoV2 S protein (Figure S3e), which confirmed the functionality of the stably integrated plasmid construct.

In the next step, we performed various fusion assays to confirm whether such localization and arrangements of S protein on these SCFM1 can successfully render the membrane fusion potential to it or not. Such fusion assay has been performed before to prove the cell-cell fusion events [12,17,18]. The efficient fusion between cells due to specific receptor–ligand interaction led to the generation of multinucleated giant cells through syncytia formation [12]. For membrane fusion assay, we chose VERO cells, which are known to express the ACE2 receptor, the specific ligand for SARS-CoV2 S protein [19]. It has been shown previously that MLV-based SARS-CoV2 pseudovirus, expressing the functional S protein on the surface, fused with VERO cells through specific interaction of S and ACE2 receptor [6].

**Figure 2.**
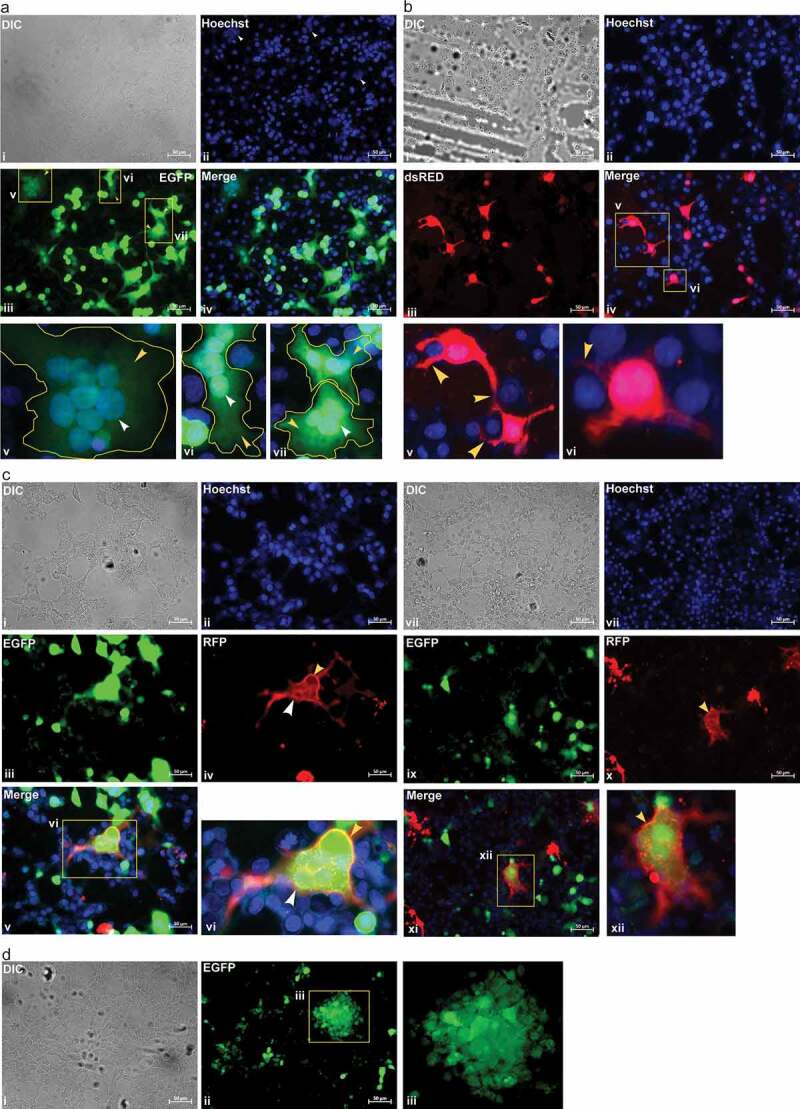
Image showing fusion of SCFM1 with target cells only, through specific interaction of ACE2 Receptor

We cultured VERO cells and generated SCFM1 followed by co-culturing them together for triggering of effective fusion through specific interaction of functional S protein, present on the surface of SCFM1 to its ligand ACE2, present on VERO cells. Our observation of cells post-fusion revealed the presence of multinucleated giant cells through syncytia formation (Figure 2(a)). As SCFM1 carries EGFP expression hence, the multinucleated giant cells showed EGFP fluorescence contributed by SCFM1. The presence of multi-nuclei was prominent through Hoechst staining (Figure 2(a)). We also put HEK293T cells transfected with pCAG-DsRed2 for fusion assay as control. We have found no syncytia formation containing multiple nuclei in this assay (Figure 2(b)). Though, in many places, we discovered that HEK293T cells were cohabiting surrounding the VERO cells but failed to fuse with them.

(a) Image showing fusion of SCFM1 with VERO cells which is evident by the generation of multinucleated giant cells through syncytium. The cytoplasm of syncytium show expression of EGFP which is contributed by the SCFM1, marked by the **yellow arrowhead**. The multinucleate formation is marked by a **white arrowhead. v., vi and viii**. show the magnified view of the area marked in the image **iv**. The nucleus was stained with Hoechst. Scale bar 50 µm.(b) Image showing co-culture of pCAG-DsRed2 transfected HEK293T cells with VERO cells. No formation of syncytia is observed, though HEK293T cells are found cohabiting surrounding the VERO cells, marked by a **yellow arrowhead. v**. and **vi**. show the magnified view of the area marked in image **iv**. The nucleus was stained with Hoechst. Scale bar 50 µm.(c) Image showing membrane fusion of SCFM1 with its target cells VERO specifically. Post fusion, a part of the membrane of the newly formed syncytium, not whole, shows an expression of RFP due to the presence of membrane tagged RFP contributed by mTagRFP-Membrane-1 transfected VERO cells, marked by a **yellow arrowhead**. Part of the membrane in the same syncytium does not show any presence of RFP which is contributed by the SCFM1 that lacks membrane-localized RFP, marked by a **white arrowhead**. The cytoplasm of such syncytium shows an expression of EGFP only, contributed by the SCFM1. **vi**. and **xii**. shows the magnified view of the area marked in the images **v**. and **xi**. The nucleus was stained with Hoechst. Scale bar 50 µm.(d) Image showing fusion of SCFM1 with MCF7 cells through specific interaction of human ACE2 (hACE2) receptor, expressed exogenously on MCF7 cells. Fusion followed by the formation of syncytia is observed specifically in exogenously hACE2 expressing MCF7 cells. The cytoplasm of syncytium show expression of EGFP which is contributed by the SCFM1. **iii**. show the magnified view of the area marked in image **ii**. The nucleus was stained with Hoechst. Scale bar 50 µm.

To confirm the membrane fusion followed by cytoplasmic intermixing of both the cell types, we repeated the fusion assay using VERO cells transfected with a different fluorescent marker expressing construct. The fusion of VERO cells, transfected with mTagRFP-Membrane-1 construct (through which the RFP is localized in the plasma membrane), with SCFM1, resulted in syncytia formation in which part of the membrane, not whole, fluorescent red due to the presence of RFP contributed by VERO cells (Figure 2(c)). Part of the membrane that did not show RFP in developed syncytia was contributed by the SCFM1 that lacked membrane-localized RFP (Figure 2(c)). The cytoplasm of such syncytium fluoresces only green due to the presence of EGFP contributed by the SCFM1 only. We also transfected VERO cells with DsRed expressing construct followed by fusion assay with SCFM1. Observation of cells post-fusion event revealed the presence of both fluorescent markers, EGFP and DsRed, in the syncytium, formed in many places (Figure S4a). These fusion assays confirmed the membrane fusion and cytoplasmic intermixing of SCFM1 with its target cells VERO.

To further confirm that SCFM1-mediated fusion of VERO cells is due to the specific interaction of S protein with its ligand ACE2 and not a passive fusion, we repeated the fusion assay of SCFM1 with MEF cells which lack ACE2 receptor. We transfected MEF cells with plasmid pCAG-DsRed before putting for fusion. Observation of cells post-fusion revealed no syncytium formation (Figure S4b). Though the MEF cells were found cohabiting with the SCFM1 in proximity, no fusion was discerned (Figure S4b). The inability of SCFM1 to fuse with MEF cells was due to the lack of presence of ACE2 on MEF cells. This data provided substantial evidence suggesting that the fusion of SCFM1 with VERO cells resulted from the interaction of the S receptor of SCFM1 with its ligand ACE2, present on VERO cells.

To prove that such fusion of SCFM1 is also feasible with cells expressing human ACE2 (hACE2) receptor, we transfected MCF7 cells with a construct carrying cDNA of hACE2 under the control of cytokeratin-18 promoter [20,21]. RT-PCR analysis confirmed the expression of hACE2 mRNA in the transfected cells, whereas, in the un-transfected cells, it was not detected (Figure S5a). MCF7 cells expressing hACE2 receptor fused with SCFM1 exogenously and formed syncytia (Figure 2(d)). No syncytia formation was observed in the fusion assay performed using the un-transfected MCF7 cells that did not express the hACE2 receptor exogenously (Figure S5b). This, straightway, proved that the fusion capability of the SCFM1 was due to the specific interaction of the S receptor, present on its surface with its ligand ACE2 receptor of human origin.

**Figure 3.**
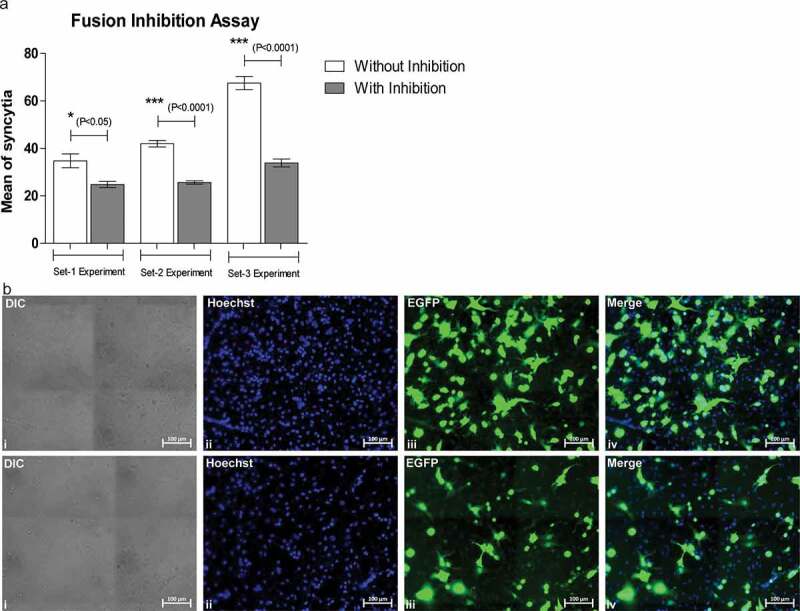
Image showing fusion inhibition assay of SCFM1 with its target VERO cells. A decrease in the number of syncitia formation was observed upon inhibition of fusion using an antibody specific for SARS-CoV2 S protein

Following this, we assessed the efficiency of the fusion and the inhibition of the fusion of the SCFM1. For this purpose, we set a fusion assay using SCFM1 incubated with an antibody specific to SARS-CoV2 S protein and native SCFM1, with VERO in different ratios (1:1 and 2:1). We observed that the number of syncytia formed was high when the fusion assay was performed with less number of SCFM1 (2 X 10^5^ in 1:1 ratio) compared to a higher number of SCFM1 (4 X 10^5^ in both 1:1 and 2:1 ratio). This proved that the SCFM1 is efficient in initiating fusion in a lower ratio also by interacting with its target receptor (Figure 3(a)). In the same experiments, when the antibody- treated SCFM1 was used, we observed a decrease in the number of syncytia formation, which indicated the inhibition of the fusion due to the lack of interaction of SARS-CoV2 S protein with its receptor ACE2, which is present on VERO cells (Figure 3(a),Figure 3(b)). Such inhibition of the fusion was uniform in various observations across the well (Figure S6 and S7). This also proved substantial evidence to suggest that this SCFM1 can be used to test the efficacy of various therapeutics in inhibiting the actual fusion of the SARS-CoV2 virus with its target cells.

(a) The bar graph showing the comparative analysis of mean of syncytia formed upon fusion of SCFM1 and VERO cells, without and with inhibition in three different sets of experiments. Inhibition of fusion followed by a decrease in the number of syncytia formation was observed upon treatment of SCFM1 with an antibody specific for SARS-CoV2 S protein. Max significant inhibition was found at set 3 experiment with p value < 0.0001. Set1 and Set2: represent inhibition assay with4 × 10^5^ SCFM1 in 1:1 and 2:1 ratios, Set3: represents inhibition assay with2 × 10^5^ SCFM1 in 1:1 ratio.(b) Upper panel of the image showing fusion of SCFM1 with VERO cells followed by the formation of syncytia. The cytoplasm of such syncytium shows an expression of EGFP contributed by the SCFM1. The nucleus was stained with Hoechst. Scale bar 100 µm. The lower panel of the image showing fusion of inhibitor (antibody specific for SARS-CoV2 S protein) treated SCFM1 with VERO cells followed by the formation of syncytia. The cytoplasm of such syncytium shows an expression of EGFP contributed by the SCFM1. The nucleus was stained with Hoechst. Scale bar 100 µm.

We have successfully generated the SCFM capable of fusion like the SARS-CoV2 virus with its host cells through specific interaction of the S receptor present on the SCFM surface with its ligand human ACE2 receptor. In this COVID19 pandemic situation, the quest for effective remedy by therapeutics or prevention of infection by vaccination has become the priority. In both the scenario, for testing the effectiveness of the newly developed therapeutics or vaccine require assay which involves the assessment of inhibition of the interaction of Spike protein of SARS-CoV2 virus with its ligand hACE2 receptor [4]. Also, antibody generated in response in vaccinated individuals needs to be tested for its efficacy in inhibiting further viral entry [22]. For this, testing a large number of samples collected at multiple time points from a large group of volunteers is necessary. For such testing large quantity of fusion-enabled pseudovirus or heat-inactivated virus particles needs to be handled in the BSL3 facility, which is time consuming, cumbersome, and costly process [23,24]. Previously researchers have developed such mammalian cells that expressed SARS-CoV2 S protein on its surface transiently for studying the membrane fusion as well as estimating its fusion efficiency.

**Figure 4.**
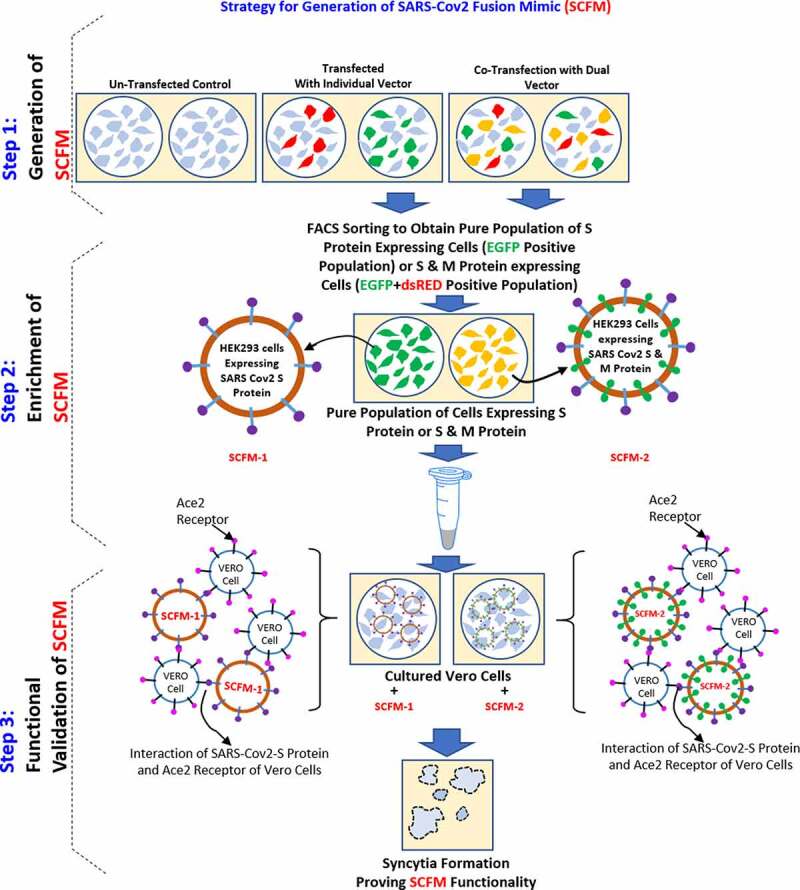
Figure describing the overall strategy for the generation of SARS-CoV2 Virus Fusion Mimic (SCFM)

But those studies have drawbacks such as transient expression of S Protein could lead to variability in fusion efficiency as well inhibition efficiency in different batches, and it is less effective as compared to pseudovirus particles [25–27]. Also, transient transfection of a huge number of cells every time will lead to cost escalation. We have developed this SCFM that can multiply while stably expressing the SARS-CoV2 S protein on its membrane and generate a platform that could be used for testing the inhibitory effect of various molecules on SARS-CoV2 membrane fusion. However, this newly developed method for the generation of fusion enabled SCFM is easy to perform in a cost-effective manner (Figure 4). The average cost for a single inhibition assay (considering technical replicate in triplicate along with control) would be much less as compared to such assay involving pseudoviruses, which are also very difficult to perform and needs stringent biological safety level practices. In the present scenario, the development of inhibitors/therapeutics against the SARS-CoV2 virus is at a fast pace [28,29]. Against this backdrop, an easy and robust platform for testing these inhibitors is the need ofthe hour. With this newly developed SCFM, such inhibition assays can be performed easily on a large scale, faster pace, and at a low cost, without the requirements of any BSL3 facility. Also, as this SCFM follow the exact mechanism of receptor-mediated fusion with the host cells, ensure the reliability of such assay.

## 4. Conclusion

As the COVID-19 has hit almost all parts of the world, including various densely populated third world countries [30], the availability of vaccines and other therapeutics at a faster pace, as well as at low cost, is the need of the hour. We have developed fusogenic mimics for the SARS-CoV2 virus, which can fuse with its target cells through the specific interaction of S protein with its receptor ACE2. We have also shown that blocking the S protein using an anti-S-protein antibody inhibits the fusion of these mimics with its target cells. We propose that this newly developed SCFM can be used as a platform to help in testing the various potential therapeutics and also for testing the efficacy of vaccines against the SARS-CoV2 virus. This method will also help in the development of vaccines and therapeutics at a faster pace with relatively low cost by easing the process as well as downsizing the requirements of cost-bearing facilities like BSL3. This strategy may help in the future for the generation of similar types of mimics for other viruses also.

## Supplementary Material

Supplemental Material

## Data Availability

All data generated or analyzed are available from the corresponding authors on request.
