# Supplementary material for: An easy method for developing fusion enabled SARS-CoV2 virus fusion mimic (SCFM), bypassing the need of Bio Safety Level (BSL) facility
Source: Bioengineered. 2021 Aug 26;12(1):4407–19. doi: 10.1080/21655979.2021.1955509 (PMC8806791; doi:10.1080/21655979.2021.1955509)
Supplement: Supplemental Material [file KBIE_A_1955509_SM0114.pdf]

Supplementary Information

Title: An easy method for developing fusion enabled SARS-CoV2 virus fusion mimic (SCFM), bypassing the need of Bio Safety Level (BSL) facility.

Authors: Abhishek Das[1], Satarupa Dutta[1], Dewanshu Sharma[1], Amit Pal[1], Nirmalya Ganguli[1,*] Subeer S. Majumdar[1,*].

Affiliation: [1]National Institute of Animal Biotechnology, Hyderabad, Telangana, India.

[*]**Corresponding Author**: Subeer S. Majumdar, Ph. D. and Nirmalya Ganguli, Ph. D.

Gene and Protein Engineering Laboratory,

National Institute of Animal Biotechnology, Hyderabad,

Telangana, India.

E. Mail: subeer@niab.org.in, nganguli@niab.org.in

Figure: S1

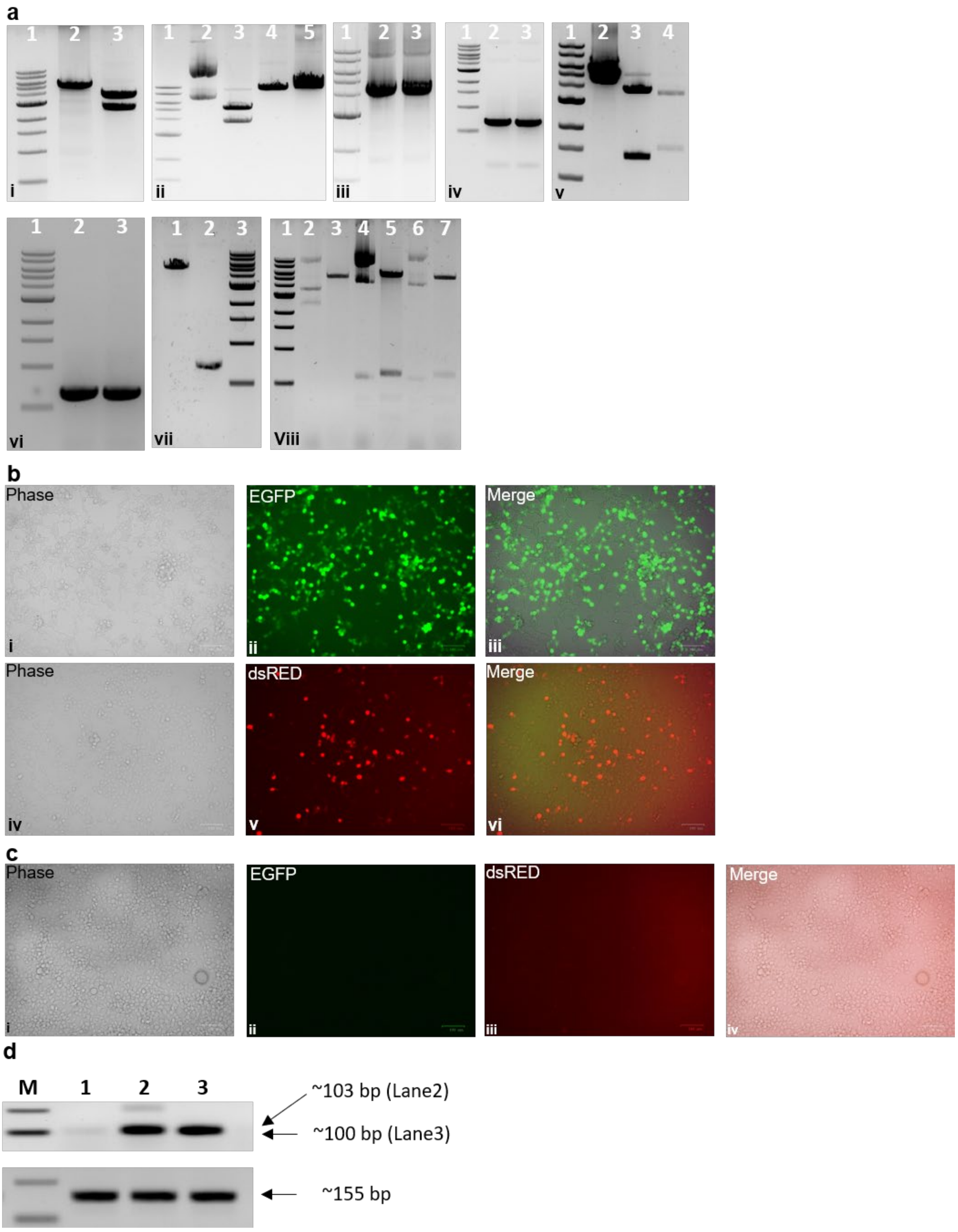

Fig. S1: Image showing generation of mammalian expression vector for SARS-Cov2 S and M protein by molecular cloning.

a. i) Agarose gel image showing digested pIRES2-EGFP and pUC57-2019-nCov-S with SacI and XmaI. Lane 1: 1 Kb DNA ladder, Lane 2: SacI and XmaI digested pIRES2-EGFP generating a linearized band of 5.2 and Lane 3: SacI and XmaI digested pUC57-2019-nCoV-S generating two bands at 3.8 Kb and 2.6 Kb position, where the upper band at 3.8 Kb position indicates human codon optimized cDNA of S protein gene. ii) Agarose gel image showing confirmation of the sequence of putative positive plasmid using restriction digestion profiling. Lane 1: 1 Kb DNA ladder, Lane 2: Undigested plasmid, Lane 3: plasmid digested with SacI and XmaI which will generate two bands at 3.8Kb position and 5.2 Kb position, Lane 4: Restriction digestion of plasmid using NotI which will yield a linearised band of size 9.1 Kb and lane 5: Restriction digestion of plasmid using AccI which will yield a linearised band of size 9.1 Kb iii)Agarose Gel image showing PCR amplification of pIRES2 backbone to remove the EGFP component from the plasmid pIRES2-EGFP. Lane 1: 1 Kb DNA ladder, Lane 2 and 3: showing amplification of pCMV-IRES2 backbone yielding a band at size 4.5Kb. iv) Agarose Gel image showing PCR amplification of DsRed2 ORF region using pCAG-DsRed2 as a template. Lane 1:1 Kb DNA ladder, Lane 2 and 3: shows amplified DsRed2 ORF of size 700 bp. v) Agarose gel image showing restriction profiling of the plasmid isolated from the colonies obtained after ligation of DsRed2 in pIRES2 bacKbone. Lane 1:1 Kb DNA ladder, Lane 2: Plasmid from self ligate control digested with enzyme StuI. Lane 3 and 4: Plasmid isolated from different colonies digested with enzyme StuI. Plasmid in lane 3 gave correct restriction digested fragments of size 3.8 Kb and 1.4 Kb position whereas in Lane 4 Restriction digestion profiling showed incorrect band pattern. vi) Agarose gel image showing PCR amplification of cDNA of M protein gene using pET-28a(+)-M protein (MC_0101136) as template. Lane 1:1 Kb DNA ladder, Lane 2 and 3: band at 693BP position shows amplification of cDNA of M protein gene from plasmid from pET-28a(+)-M protein (MC_0101136). vii) Agarose gel image showing digested pIRES2-DsRed2 M protein gene using SacI and XmaI, Lane 1: Digested pIRES2-DsRed2, Lane 2: Digested PCR amplified M protein encoding ORF, Lane 3: 1 Kb DNA ladder. viii) Agarose gel image showing restriction profiling of the plasmid isolated from the colonies obtained after ligation of cDNA of M protein gene in pIRES2-DsRed2 backbone. Lane 1: 1Kb DNA ladder, Lane 2: Undigested plasmid from self-ligate control colony, Lane 3: Plasmid from self-ligate control colony digested with restriction enzymes AgeI and NdeI generates a single band at 5.2 Kb position. Lane 4 and 5: Indicates undigested and plasmid digested with AgeI and NdeI respectively where a lower band at 622 Bp position and an upper band at little above than 5.2 Kb position indicates absence of positive clone. Lane 6 and 7:

Indicates undigested plasmid and plasmid digested with AgeI and NdeI shows correct band pattern at 622BP position and at 5.2 Kb position which confirms positive clone.

b. Images showing expression of EGFP or DsRed2 inHEK293T cells transfected with constructs pCMV-SARSCoV2S-IRES2-EGFP or pCMV-SARSCoV2M-IRES2-DsRed2 respectively. Images (i), (ii) and (iii) show the transfected cells with pCMV-SARSCoV2S-IRES2-EGFP. Images (iv), (v) and (vi) shows the transfected cells with pCMV-SARSCoV2M-IRES2-DsRed2.

c. Images showing untransfected HEK293T cells when visualised under microscope with different UV filters.

d. Image showing detection of transcripts of S and M protein from total RNA isolated from HEK293T cells transfected with pCMV-SARSCoV2S-IRES2-EGFP and pCMV-SARSCoV2M-IRES2-DsRed2 respectively. Human beta-actin expression is estimated as the house keeping gene control; Lane 1 denotes Untransfected HEK293T cells, Lane 2 and 3 denotes pCMV-SARSCoV2S-IRES2-EGFP and pCMV-SARSCoV2M-IRES2-DsRed2 transfected HEK293T cells, respectively. Pictures have been cropped to size for suitable representation.

Figure: S2

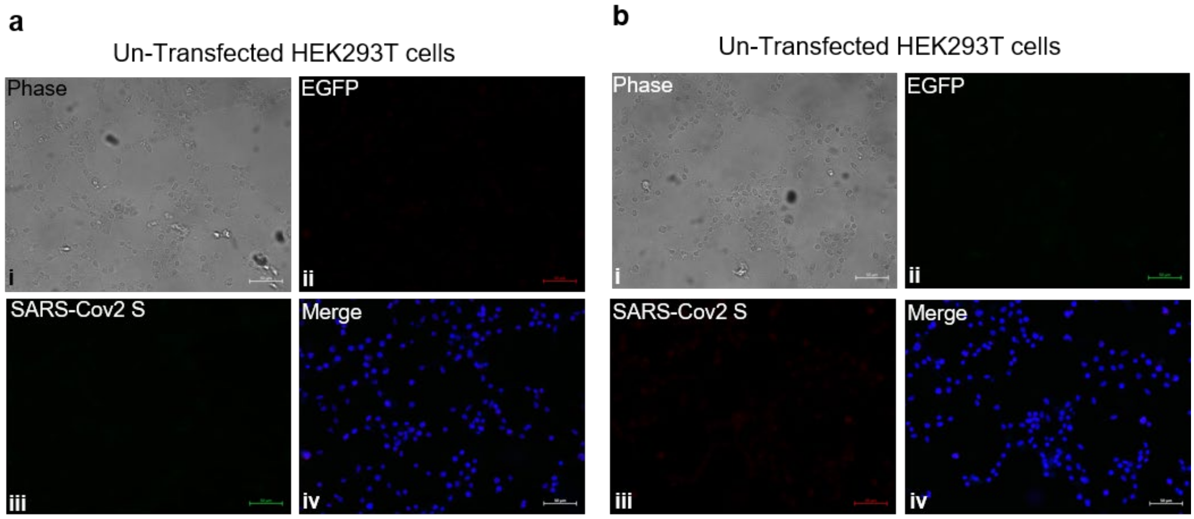

Fig. S2: Image showing immuno cytochemistry analysis of untransfected HEK293T cells treated with SARS-CoV2 S protein specific antibody followed by counter stained with Alexaflour 546 secondary antibody.

a. No expression of EGFP as well as SARS-CoV2 S protein was observed in the un-transfected cells upon treatment with antibodies in permeabilised state. **i.** Represent the phase contrast images. **ii.** Shows the image captured under UV illumination with FITC filter (for EGFP). **iii.** Shows the image captured under UV illumination with TRITC filter (for AF546). **iv.** Shows the merged images. Nucleus was stained with Hoechst, captured under UV illumination with Blue filter and merged. Scale bar 50µm.

b. No expression of EGFP as well as SARS-CoV2 S protein was observed in the un-transfected cells upon treatment with antibodies in non-permeabilised state. **i.** Represent the phase contrast images. **ii.** Shows the image captured under UV illumination with FITC filter (for EGFP). **iii.** Shows the image captured under UV illumination with TRITC filter (for AF546). **iv.** Shows the merged images. Nucleus was stained with Hoechest, captured under UV illumination with Blue filter and merged. Scale bar 50µm.

Figure: S3

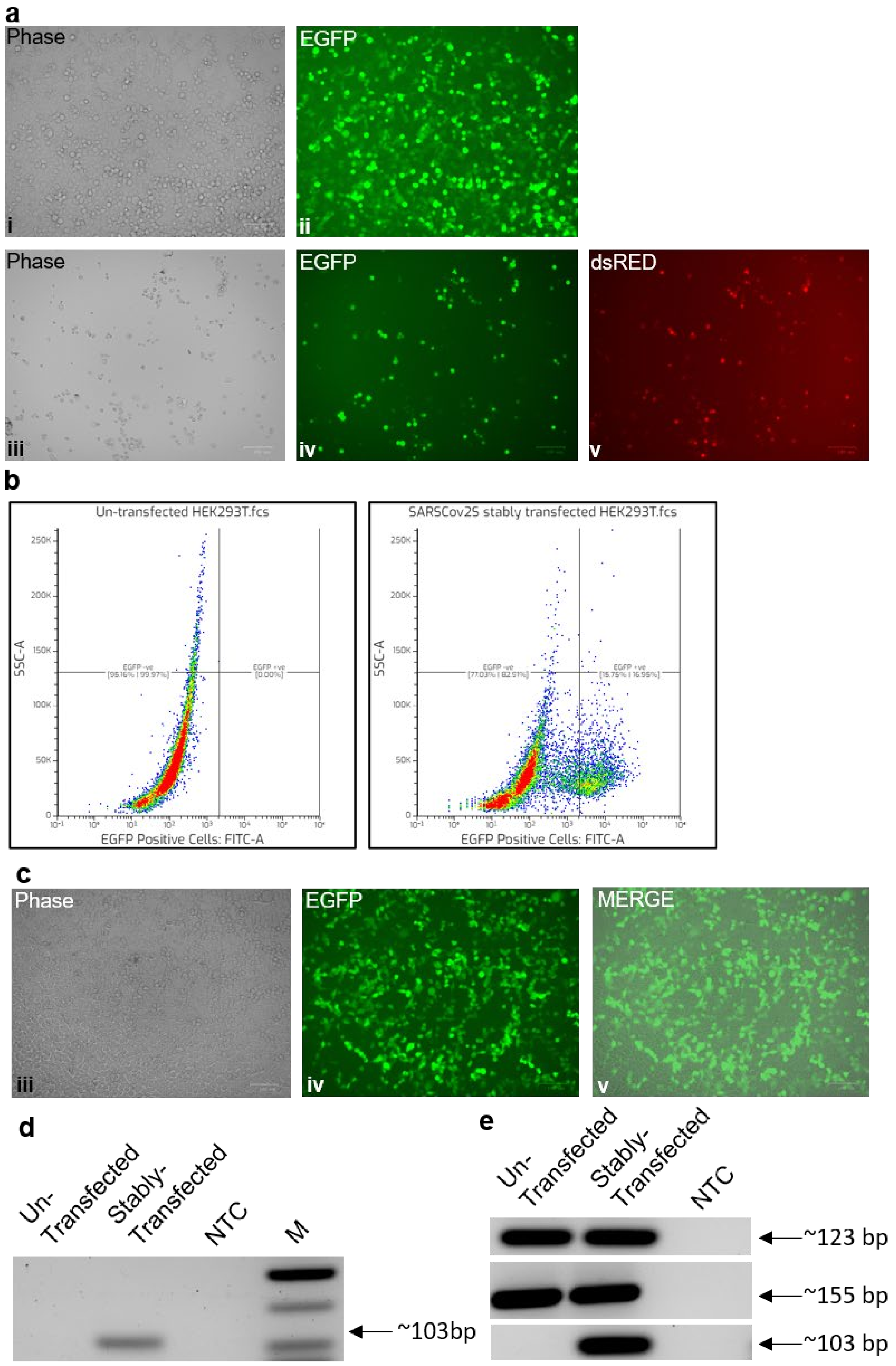
a
Phase
EGFP
i
ii
Phase
EGFP
dsRED
iii
iv
v
b
Un-transfected HEK293T.fcs
SARSCov2S stably transfected HEK293T.fcs
SSC-A
EGFP Positive Cells: FITC-A
c
Phase
EGFP
MERGE
iii
iv
v
d
Un-Transfected
Stably-Transfected
NTC
M
~103bp
e
Un-Transfected
Stably-Transfected
NTC
~123 bp
~155 bp
~103 bp

Fig. S3: Image showing generation of HEK293T cells stably expressing the SARS-CoV2 S protein on its surface – SCFM1.

a. Image showing observation of EGFP and DsRed2 expression in cultured HEK293T cells transfected with expression vector bearing cDNA of SARS-Cov2 S and M protein. Cells were sorted through FACS before culturing. Upper panel showing expression of EGFP in the cells transfected with pCMV-SARS-CoV2S-IRES2-EGFP expression vector. Lower panel showing expression of EGFP and DsRed2 both in the cells co-transfected with pCMV-SARS-CoV2S-IRES2-EGFP and pCMV-SARS-CoV2M-IRES2-DsRed2 expression vector. **i.** and **iii.** Represent the phase contrast images. **ii.** and **vi.** Shows the image captured under UV illumination with FITC filter (for EGFP). **v.** Shows the image captured under UV illumination with TRITC filter (for DsRed2). Scale bar 100µm.

b. Densitometry graph showing the Flow cytometry analysis of the HEK293T cells at passage #14 stably transfected with pCMV-SARSCoV2S-IRES2-EGFP construct; First plot represents un-transfected HEK293T cells, and second plot represents pCMV-SARSCoV2S-IRES2-EGFP construct stably transfected HEK293T cells.

c. Image showing observation of EGFP expression in cultured HEK293T cells stably transfected with pCMV-SARSCoV2S-IRES2-EGFP construct at passage #17. **i.** Shows the phase contrast image. **ii.** Shows the image captured under UV illumination with FITC filter (for EGFP). **iii.** Shows the merged image.

d. Agarose gel image showing detection of S-protein gene amplified from genomic DNA isolated from HEK239T cells stably transfected with pCMV-SARSCoV2S-IRES2-EGFP construct. Pictures have been cropped to size for suitable representation.

e. Agarose gel image showing detection of transcript of S-protein from total RNA isolated from HEK293T cells stably transfected with pCMV-SARSCoV2S-IRES2-EGFP construct. Human GAPDH (upper panel) and beta-actin expression (middle panel) were estimated as the housekeeping gene control; Lower panel shows the amplification of the transcript of SAR-CoV2 S protein in the stably transfected cells only. Pictures have been cropped to size for suitable representation.

Figure: S4

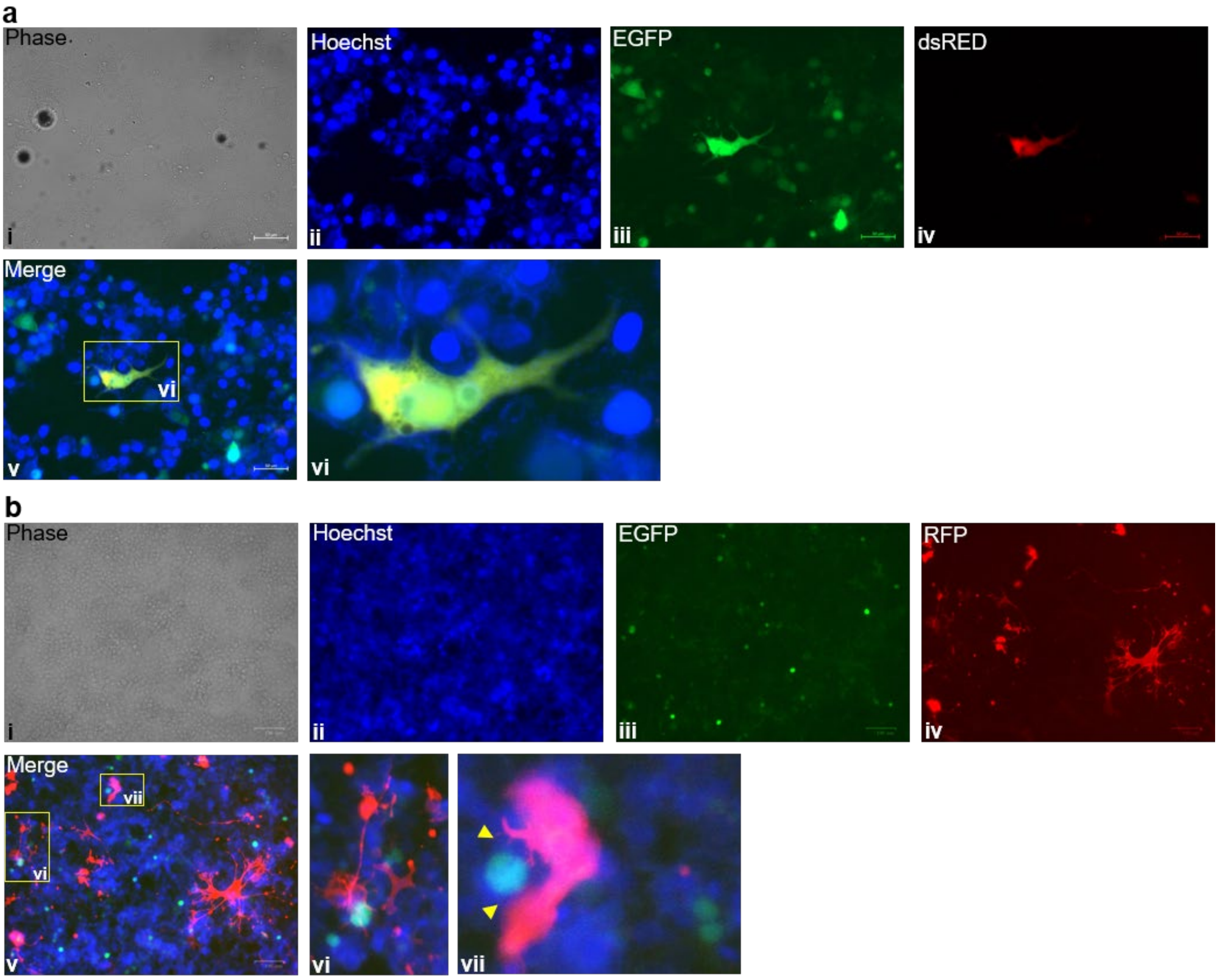

Fig. S4: Image showing cytoplasmic intermixing of SCFM1 and VERO cells upon membrane fusion.

a. Image showing fusion followed by cytoplasmic intermixing of SCFM1 with cultured VERO cells, transfected with pCAG-DsRed2. The fused cytoplasm of the developed syncytia showed expression of both EGFP as well as DsRed2 protein contributed by SCFM1 and pCAG-DsRed2 transfected VERO cells respectively. **i.** Represent the phase contrast images. **ii.** Shows the image captured under UV illumination with blue filter (for Hoechst). **iii.** Shows the image captured under UV illumination with FITC filter (for EGFP). **iv.** Shows the image captured under UV illumination with TRITC filter (for DsRed2). **v.** Shows the merged images. **vi.** shows the magnified view of the area marked in the image **v.** Nucleus was stained with Hoechst. Scale bar 50 µm.

b. Image showing coculture of SCFM1 and mouse embryonic fibroblast cell (MEF). No syncytia formation was observed despite the MEF cells are found cohabiting with the SCFM1

in proximity attached to each other but without any fusion event, marked by **yellow arrowhead**. **i.** Represent the phase contrast images. **ii.** Shows the image captured under UV illumination with blue filter (for Hoechst). **iii.** Shows the image captured under UV illumination with FITC filter (for EGFP). **iv.** Shows the image captured under UV illumination with TRITC filter (for DsRed2). **v.** Shows the merged images. **vi.** and **vii.** shows the magnified view of the area marked in the image v. Nucleus was stained with Hoechst. Scale bar 50µm.

Figure: S5

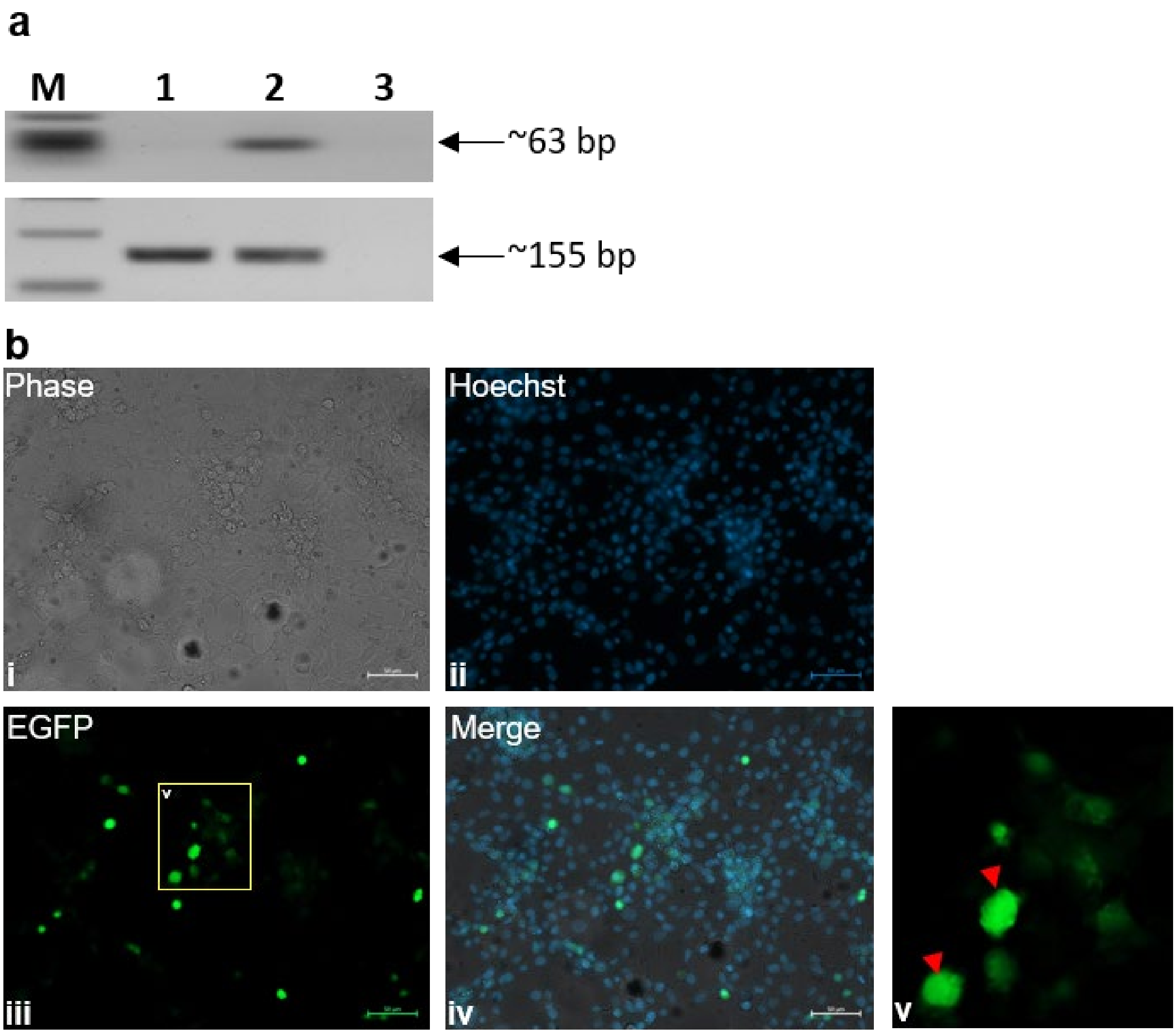

Fig. S5:

a. Detection of expression of hACE2 receptor in MCF7 cells upon transfection with hACE2 bearing plasmid DNA. Image showing agarose gel electrophoresis of RT-PCR performed using total RNA extracted from MCF7 cells transfected with hACE2 expressing vector along with untransfected cells. Expression of hACE2 transcripts were specifically detected in the transfected cells as compared to untransfected cells. Upper panel show amplification of hACE2 mRNA (~63bp) and lower panel show amplification of beta-Actin mRNA as housekeeping control (~155 bp). **M.** denotes NEB Lowmolecular weight DNA marker (upper panel) & 100bp molecular weight marker (Lower panel). **1.** Denotes total RNA isolated from untransfected MCF7 cells. 2**.** Denotes total RNA isolated from hACE2 cDNA bearing expression construct transfected MCF7 cells. **3.** Denotes non-template control (NTC).

b. Image showing coculture of SCFM1 and wildtype MCF7 cells. No syncytia formation is observed due to lack of hCAE2 expression on wildtype MCF7 cells. **i.** Represent the phase contrast images. **ii.** Shows the image captured under UV illumination with blue filter (for Hoechst). **iii.** Shows the image captured under UV illumination with FITC filter (for EGFP). **iv.** Shows the merged images. **v.** shows the magnified view of the area marked in the image iii. Nucleus was stained with Hoechst. Scale bar 50μm.

Figure: S6

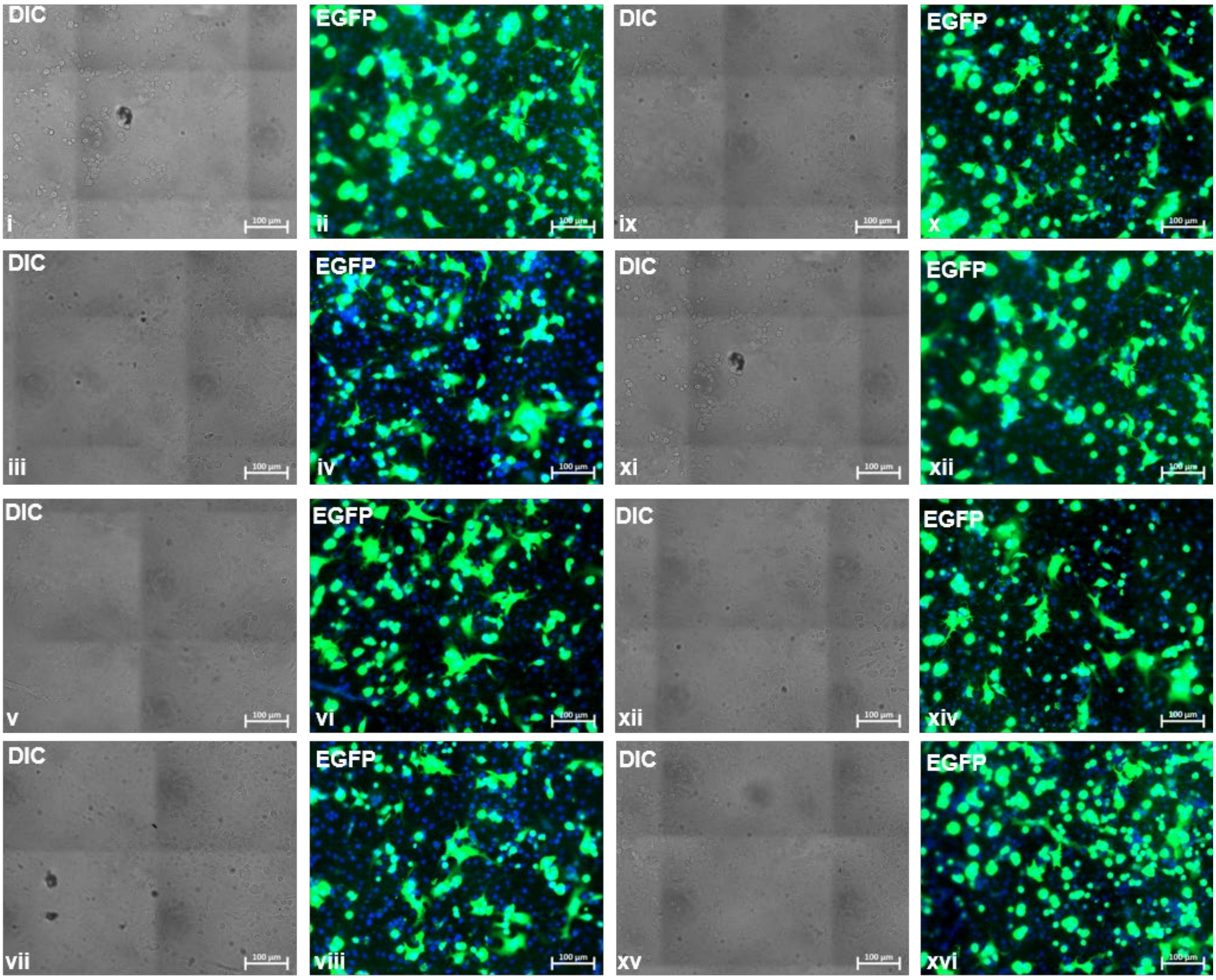

Fig. S6: Image showing fusion assay of SCFM1 with target VERO cells without antibody inhibition. Images from different fields were taken which show syncytia formation leading to generation of multinucleated giant cell formation in all the images. Nucleus staining was done by using Hoechst dye. EGFP expression was contributed by the SCFM1. Scale bar 100 µm.

Figure: S7

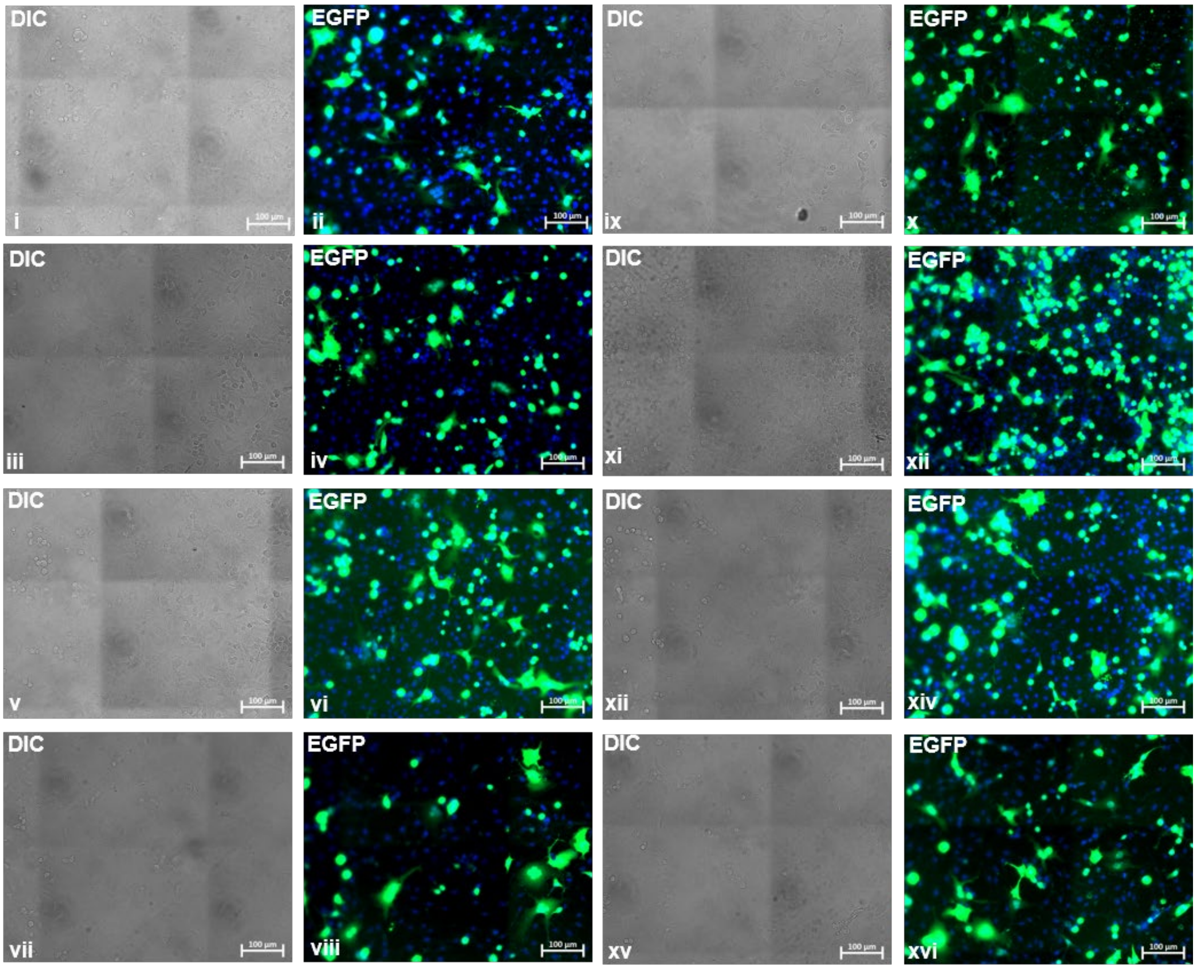

Fig. S7: Image showing fusion assay of SCFM1 with target VERO cells with antibody inhibition. The SCFM1 cells were preincubated with Anti Spike antibody prior to fusion. Images from different fields were taken which show syncytia formation leading to generation of multinucleated giant cell in all the images. These images clearly show decrease in syncytia formation as compared to control set (Supplementary figure S6). EGFP expression was contributed by the SCFM1. Nucleus staining was done by using Hoechst dye. Scale bar 100 µm
